# Workbench for a Parabolic Trough Solar Collector with a Tracking System

**DOI:** 10.1155/2022/4505349

**Published:** 2022-07-05

**Authors:** Luciano A. Fiamonzini, Gustavo A. R. Rivas, Oswaldo H. Ando Junior

**Affiliations:** ^1^Programa de Pós-Graduação Interdisciplinar em Energia e Sustentabilidade (PPGIES), Universidade Federal da Integração Latino Americana (UNILA), Foz do Iguaçu, Brazil; ^2^Unidade Acadêmica do Cabo de Santo Agostinho (UACSA), Universidade Federal Rural de Pernambuco (UFRPE), Recife, Brazil

## Abstract

Solar energy found abundantly in nature is considered a renewable energy source. It is also of great interest as an option for energy generation and CO_2_ emissions reduction. Several technologies of solar concentrating systems, known internationally as CSP (concentrated solar power), are found in the industrial and scientific environment. One of the most mature and internationally known technologies is the parabolic trough solar collector (PTSC), which has several applications, such as electricity generation, desalination, steam generation, and refrigeration systems, among others. However, more research and development (R&D) has been done to improve its performance, using new materials, absorber tube geometries, solar tracking systems, and work (thermal oils, nanofluids). Thus, the present work describes the development of a low-cost PTSC for academic and research purposes. The PTSC was built with an edge angle of 120°, an opening area of 2.2 m^2^, and a copper absorber tube of 42 mm in outer diameter without a glass envelope. The gutter structure is composed of wooden sheets cut in a parabolic shape, where a 1.2 mm-thick galvanized steel sheet coated with a reflective film is supported, thus functioning as the reflective surface of the PTSC. The solar tracking system is one of the active types with two axes containing photoresistive sensors, which are used to determine the solar position and electric actuators to correct the positioning of the gutter. The monitoring system was developed through an interactive panel to visualize the operating parameters of the sensing elements, thermocouples that measure the inlet and outlet temperature in the absorber tube, and the flow sensor to measure the flow of the heat transport fluid. Laboratory tests were performed with deionized water as a transport fluid, establishing two testing conditions. The first test condition analyzed the efficiency of the collector at different temperatures. Thus, the inlet temperature varied, between 30 and 70°C, presenting a flow of 0.020 kg/s. The second one evaluated the collector efficiency for different flows, subjecting the collector to flows from 0.002 to 0.030 kg/s. Thus, the proposed collector obtained an efficiency as a function of the temperature represented by the expression *η* = 0.324–2.47443 *c*′, where *c*′ is a parameter that relates the inlet temperature to the ambient temperature as a function of the solar radiation available. Yet, the efficiency in function of the flow became optimal when the flow regime became turbulent. It was concluded that the proposed solar collector obtained lower efficiency when compared with other collectors in the literature, which was assumed to be due to the diffusion losses of the parabolic trough reflector and thermal losses by convection in the parabolic trough absorber tube (optical efficiency, removal factor, and heat loss coefficient).

## 1. Introduction

**Figure 1:**
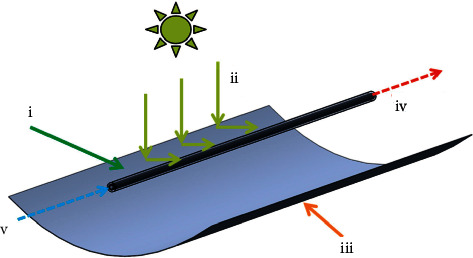
Representation of a PTSC: (i) absorber tube, (ii) solar rays, (iii) parabolic trough, (iv) fluid outlet temperature, and (v) fluid inlet temperature [4].

The energy from solar radiation is considered an abundant and inexhaustible energy resource [1]. The parabolic trough solar collector (PTSC) is among the main technologies of solar concentration, internationally known as CSP (concentrated solar power). It is one of the most mature energy sources with great applicability in the energy segment. In this PTSC technology (see Figure 1), solar radiation is converted into heat. The PTSC consists of a parabolic trough (iii) exposed perpendicularly to the normal vector of solar radiation (i). Its shape promotes the distribution and concentration of solar rays in a focal line. In this position, the component called the absorber tube (ii) receives solar radiation, converting it into heat, which is transmitted to the heat transfer fluid (HTF) [2, 3].

PTSCs have a reflective film adhered to the surface of their parabolic trough, which is manufactured in curved glass mirrors. This surface must have mechanical strength and good quality optical properties for its application. In constructive terms, this component is considered expensive and complex. However, low-cost alternatives seeking to improve the constructive process of this component are constantly proposed. As previously mentioned, the solar rays concentrated on the curved surface are directed to the absorber tube, which basically consists of a metallic tube with an absorber surface that receives solar radiation and conducts the absorbed heat to a heat transport fluid [5]. For these two components (absorber tube and reflective surface) to function properly, it is necessary to implement auxiliary systems such as a system to control the flow, temperature, and operating conditions of the PTSC; a tracking system, responsible for the parabolic trough movement and solar tracking; and finally, a circulation and storage system for the heat transport fluid [6].

The parabolic trough can be considered the main part of the system, in which a curved glass mirror was widely used due to its high efficiency. However, the construction of this mirror was expensive because the more accurate the curved geometry is, the higher is its production cost. However, silver-coated plastic films, anodized aluminum sheets, and aluminum foil sheets can be considered low-cost alternatives. Therefore, the study carried out by Sagade and Aher et al. [7] tested an aluminum coating for the reflector where their experimental model achieved an efficiency of 59.8% with a mass flow of 0.011 kg/s, where the efficiency in different situations of ambient temperature and wind incidence were compared.

A PTSC necessarily needs some devices to operate appropriately, such as a pump for the forced circulation of heat transfer fluid (FTC), a temperature and flow monitoring system, and also a solar tracking device, which maintains the correct perpendicularity between the gutter and the sun rays. Solar tracking systems can be active, where a motor is usually associated with a computer, which consists of a program that adjusts the orientation of the collector according to the date and time of the day. There are also passive ones, which heat a liquid integrated into the structure of the system's support, changing its center of gravity, thus, rotating the structure which follows the Sun [8].

In turn, active tracking systems can have one or two axes. Single-axis trackers can be applied to PTSCs and linear Fresnel-type collectors. The two-axis tracking can be applied to parabolic dish solar collectors (PDSCs), solar concentration towers, and also to the PTSC. Note that solar tracking allows the energy captured to increase in the range of 30 to 40% compared with systems without solar tracking [8].

Among the PTSC concentrator collectors with tracking on only one axis, they are considered as devices with very mature technology. However, the development of new materials, mechanisms, or processes that reduces the cost of implantation and maintenance or even increases efficiency and availability must be researched. To meet this need, the development of a didactic workbench of two PTSC axes was proposed [3].

Concerning the construction of a low-cost PTSC, Fathabadi [9] used a closed thermosiphon, a two-phase collector tube; while, for the reflective surface, he used a polished stainless-steel plate. Fathabadi compared the manufacturing cost of its collector with the commercial price of the competitor's collectors to prove its low cost and arrived at a Euro/W ratio, where the proposed collector rate was 0.4467 Euro/W, while the evacuated tube collector cost was 0.8364 Euro/W. It was not cheaper than the flat plate collector, which costs 0.344 Euro/W.

The thermal use of solar energy through thermosolar systems such as the PTSC is a viable alternative. However, to promote the advancement of this technology, it is necessary to conduct experimental studies that prove the benefits of these advances. So, the development of this didactic board is a way to provide support for future research related to this area. Following the line of the authors previously mentioned, the present work developed a low-cost mobile-teaching bench composed of a parabolic trough solar collector (PTSC). This teaching bench can analyze the thermal performance of different heat transport fluids under different working conditions. Also, it can be configured with tubes of different geometries that can improve its performance. Furthermore, it aims to develop a system built using alternative and low-cost materials.

### 1.1. PTSC Design and Construction

Regarding the materials and construction method, physical and mechanical properties must be reconciled, such as weight of the structure, torsional rigidity, and resistance to weather and operating stress, allied to low operating and construction costs.

According to Thomas and Guven [10], to design a PTSC, firstly the parabolic trough must be designed and then its geometry should be determined. Thus, several parameters will be chosen and determined, such as aperture angle, aperture length, focal length, and other characteristics related to the geometry of this collector component.

Thus, the project starts by determining the parabolic profile, which can be obtained by applying equation (1), which represents a parabola.

(1) $$\begin{array}{c} x^{2}=4yf_{L}, \end{array}$$

where *x* and *y* represent the Cartesian coordinates of the surface of the parabola, and (*f*_*L*_) represents the focal distance between the center of the absorber tube and the valley of the parabola, according to equation (2).

(2) $$\begin{array}{c} f_{L}=\frac{w_{a}}{4\text{tan}\left(\emptyset_{r}/2\right)}, \end{array}$$

where (*w*_*a*_) is the length of the collector opening and (∅_*r*_) is the collector edge angle. Finally, the concentration (*C*) factor of the relationship between the collector opening area and the absorber tube area is as follows:

(3) $$\begin{array}{c} C=\frac{w_{a}}{\pi D}. \end{array}$$

**Figure 2:**
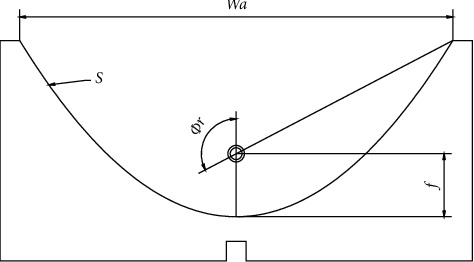
PTSC geometric parameters (see Table 1).

The geometric parameters of this collector are shown in Table 1 and Figure 2.

Regarding the absorber tube, it was decided that the material would be made of copper due to its higher value of thermal conductivity, 398 W/m^2^K [11]. This is easily acquired and widely used in hydraulic applications. A 42 mm diameter tube was chosen as it provides a greater acceptance angle, reducing the need for a high-precision solar tracking system. It should be noted that an absorber tube with a glass envelope guarantees greater efficiency for the collector [12]. However, in this study, this evacuated absorber was not used to reduce the cost of the equipment, which would present greater efficiency. The absorber tube lining normally consists of low-emissivity, high-absorbent material. However, high-temperature-resistant black matte paint was used in this project. It has an absorbance of approximately 97% and an emissivity of around 90%; it means that part of the heat absorbed is lost by irradiation. Another unfavorable point is that black ink has the lowest thermal conductivity, which increases the temperature gradient between the outer and inner surfaces of the tube [13].

**Figure 3:**
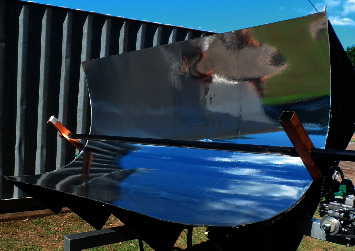
Proposed parabolic trough.

Five wooden plates were used to make the parabolic trough following the shape and dimensions of the parabola previously calculated. On the other hand, a CNC programmable cutter was used to facilitate and improve cutting precision. The cut plates were set on a metallic tube and fixed using angle brackets forming a base in the parabolic format shown in Figure 3, where it was possible to set a metallic plate of 1.2 mm of thickness fixed in wooden plates using epoxy glue and screws.

Curved mirrors or polished metals such as aluminum and stainless steel are usually used for the reflective surface. These surfaces generally achieve reflectivity values greater than 95% [6, 14]. Digrazia and Jorgensen [15] already evaluated the durability of the application of reflective films. As it is a material that is easier to mold than glass, a reflective film with 96% reflectivity was chosen (see Figure 3).

**Figure 4:**
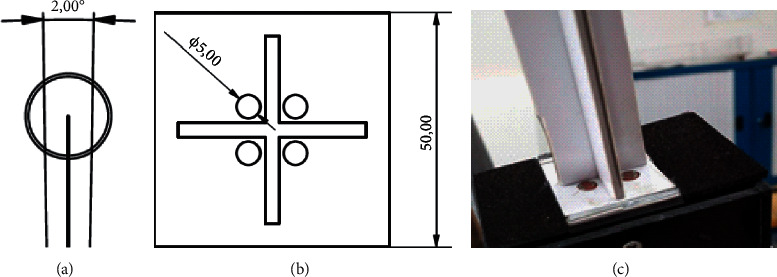
Solar positioning sensor: (a) tracking error, (b) top view of the sensor, and (c) positioning sensor.

In addition, the solar tracking and gutter movement systems were developed through a two-axis active tracking system, consisting of (1) a solar position sensor, which uses the photoresistive effect of LDRs (light-dependent resistors); (2) a controller with relay module based on an open-platform development model (Arduino); (3) Azimuthal actuator, which performs a rotation movement of the chute on the axis parallel to the absorber tube, and we used a reducer in this actuator to increase the motor torque; (4) an altitude actuator that performs the tilting movement of the gutter to follow the seasonal variation of solar inclination; and to execute such a movement, a linear actuator with a screw and (5) a solar positioning sensor were designed with a set of 4 LDRs mounted on the support and glued to the 5 mm holes, being perpendicular to the dividing tower, as shown in Figure 4. An error greater than 2.3°in the angle of incidence was noticed. The sensor must be completely shaded, a limit condition in which the correction of the positioning of the parabola should occur. For its construction, an ABS part was made using a 3D printer.

The actuators were driven by 12 V direct current electric motors used in car-door glass-lifting mechanisms. They were chosen because they have an approximate torque of 10 Newtons.

The tracking system suffers a 2° error regarding the middle of the absorber tube (see Figure 4(b)). This error occurs because the positioning sensor should be at least partially shaded to initiate a correction. For each correction, the system jumps 1°, so two jumps are required for each correction. However, this tracking error does not interfere with the incidence of concentrated rays because of the diameter of the absorber tube.

**Figure 5:**
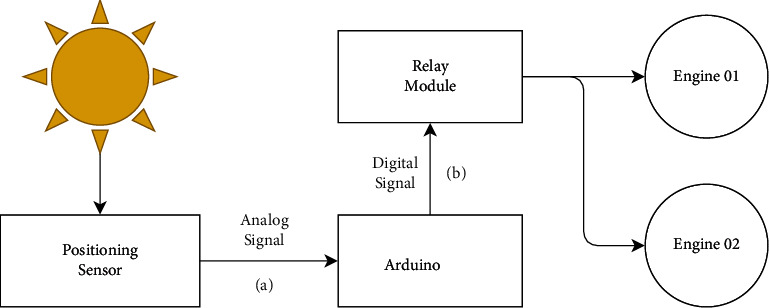
Flowchart of the operation of the solar tracking system.

As shown in Figure 5, this tracking system operates as follows: the positioning sensor LDRs vary their output voltage depending on the light falling on the surface of each LDR, so if the four LDRs are exposed to the Sun, the voltage value read by the Arduino is similar for the four LDRs. However, if one of them is shaded, the voltage level of this sensor will rise, and through the developed algorithm, it will activate the relay corresponding to the direction of the rotation necessary to correct the lag of the collector's normal plane in relation to the angle of solar incidence.

#### 1.1.1. Monitoring System

**Figure 6:**
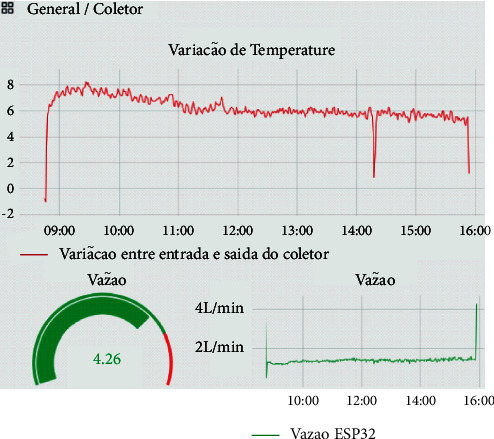
Screenshot of the data visualization panel.

The PTSC monitoring system is responsible for collecting the collector's operating data and making them available in a graphical way. Thus, the following data were collected: temperature data at the inlet and outlet of the absorber tube; mass flow rate of the transport fluid, and the external temperature of the absorber tube.  Figure 6 shows a panel created to display the operating conditions of the PTSC in real time.

**Figure 7:**
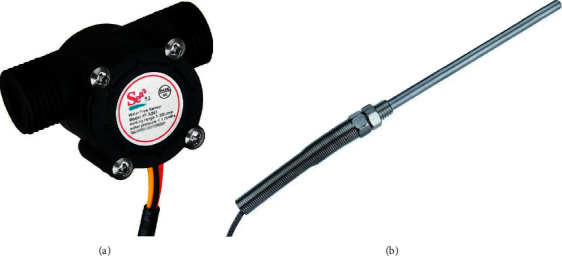
(a) Temperature sensor. (b) Flow sensor.

Type K thermocouples were used to measure temperatures (Figure 7(a)). They can operate from 0 to 1260°C, and in the range from 0 to 100°C. They have an error of less than 1%; therefore, they are used in the present laboratory experiment. An integrated circuit of the max6675 model was used as a cold junction compensation, which has a 12-bit digital output, providing a resolution of 0.25°C, with an error of 0.5°C for temperatures from 0 to 1024°C [16].

For the flow measurement, a flow sensor was applied right after the circulation pump, the sensor model used was YF-S201 (Figure 7(b)), being able to operate in a flow range from 1 to 30 L/min, and the internal measurement sensor is of the hall effect type, where a magnet fixed to one of the internal blades of the microturbine present in the sensor, switches the digital output from a low-to-high logic state, generating a response signal at each turn of the microturbine [17].

### 1.2. PTSC Methodology and Sizing

The collector time constant parameter (C_t) was used to ensure that the collected data obtained experimentally fall in the permanent or steady state. This value corresponds to the time required for the collector to reach 63.2% of the maximum constant temperature. The procedure to determine this time consists of covering the reflective surface of the collector, establishing a continuous flow of fluid, discovering the collector, and starting the time counting until the temperature variation at the collector outlet is less than 0.05°C per minute. Determining this time interval is necessary to correctly evaluate the collector efficiency because in transient regimes the efficiency can be underestimated due to the heat transfer to the collector vicinity [3].

The collector tests were performed under a steady state and for two test conditions. Then, in the first one, the flow of the heat transport fluid varied between 5 and 70 kg/h, and the inlet temperature was set at 70°C. The second test was performed with a fixed flow rate of approximately 70 kg/h at different inlet temperatures of the heat transfer fluid. As previously described, we used deionized water as fluid for both tests. The tests were performed from 08/03/2020 to 08/06/2020 between 9 : 30 and 13 : 00, with clear skies and an average ambient temperature of 25°C, where the collector was aligned in the direction of the north-south axis and located at coordinates S 25.43816, O 54.59679.

According to Kalogirou [3], the efficiency of the solar collector (*η*_*e*_) can be measured experimentally using equation (4), which is the ratio between the energy gain (*Q*_*u*_) and the available solar radiation (*Q*_*s*_) obtained with the data of direct solar radiation (*I*_*b*_) and the collector opening area (*A*_*a*_).

(4) $$\begin{array}{c} \eta_{e}=\frac{\left[\dot{m}C_{p}\left(T_{s}-T_{e}\right)\right]}{\left[A_{a}I_{b}\right]}=\frac{Q_{u}}{Q_{s}}. \end{array}$$

In equation (5), we calculate the value of the theoretical yield *η*_*t*_ using an energy balance in the receiver:

(5) $$\begin{array}{c} \eta_{t}=\frac{A_{r}F_{r}\left[\eta_{o}I_{b}-\left(U_{L}/C\right)\left(T_{e}-T_{a}\right)\right]}{\left[A_{a}I_{b}\right]}, \end{array}$$

where (*η*_*o*_) is the optical efficiency of the collector calculated as shown in equation (6) and (*ρ*) is the reflectance of the reflective surface, (*τ*) transmissivity factor of the glass envelope, (*λ*) absorption factor of the tube, (*γ*) interception factor, (A_f) geometric factor, and *θ* the angle of incidence

(6) $$\begin{array}{c} \eta_{0}=\rho \tau \lambda \gamma \left[\left(1-A_{f}\text{tan}\left(\theta \right)\text{cos}\left(\theta \right)\right)\right]. \end{array}$$

An alternative way to determine the thermal efficiency is to construct a graph that relates the collector efficiency and equation (4), with a representative heat parameter (*c*′). Thus, the thermal efficiency is now represented by a linear equation, where the interception of this line on the axis of global efficiency represents the parameter (*b*′) and the slope corresponds to the parameter (*a*′), making it possible to determine this optical efficiency using equation (7):

(7) $$\begin{array}{c} a^{\prime }=F_{r}\eta_{o}, \end{array}$$

(8) $$\begin{array}{c} b^{\prime }=\left(\frac{F_{r}U_{L}}{C}\right). \end{array}$$

(9) $$\begin{array}{c} c^{\prime }=\left(\frac{T_{i}-T_{a}}{I_{b}}\right), \end{array}$$

(10) $$\begin{array}{c} \eta_{t}=a^{\prime }+b^{\prime }c^{\prime }. \end{array}$$

Thus, determining the thermal efficiency becomes a linear equation in terms of equation (10), so it is necessary to obtain several measures of efficiency through equation (4) with different values of inlet temperature [2, 18].

Thus, to determine the optical efficiency, the parameters (F_r) should be calculated according to equation (11):

(11) $$\begin{array}{c} F_{r}=\frac{\dot{m}C_{p}}{A_{ri}U_{L}}\left[1-\text{exp}\left(-\frac{U_{L}F^{\prime }A_{ri}}{\dot{m}C_{p}}\right)\right], \end{array}$$

and the global heat transfer coefficient (*U*_*o*_) is obtained according to equation (12), where *k* is the thermal conductivity value of the tube:

(12) $$\begin{array}{c} U_{o}={\left[\frac{1}{U_{l}}+\frac{D_{e}}{h_{f}D_{i}}+\frac{D_{e}\text{ln}\left(D_{e}/D_{i}\right)}{2k}\right]}^{-1}=\frac{U_{L}}{F^{\prime }}, \end{array}$$

where (*F*′) is rewritten as equation (13):

(13) $$\begin{array}{c} F\prime =\;\frac{1/U_{l}}{\left(1/U_{l}\right)+\left(D_{e}/\left(h_{f}D_{i}\right)\right)+\;D_{e}\;\left(\text{ln}\left(D_{e}/D_{i}\right)/2k\right)}. \end{array}$$

In equation (14), (*U*_*L*_) represents the coefficient of thermal loss that can be described as the sum of the losses by convection, conduction, and irradiation:

(14) $$\begin{array}{c} U_{L}=h_{\text{amb}}+h_{r}+h_{c}. \end{array}$$

By means of equation (15), it is possible to calculate (*h*_amb_), and by means of (*h*_*r*_), equation (16), the coefficient of conduction losses will be neglected when *σ* = 5.67 × 10^−8^.

(15) $$\begin{array}{c} h_{\text{amb}}=4v_{\text{vent}}^{0,58}D_{e}^{-0,42}, \end{array}$$

(16) $$\begin{array}{c} h_{r}=4\sigma \varepsilon T_{\text{et}}^{3}. \end{array}$$

The emissivity varies according to the coverage of the absorber tube and the average temperature of the receiver (equation (17)):

(17) $$\begin{array}{c} \varepsilon =0,062+2\times 10^{-7}\times T_{o}^{2}. \end{array}$$

To obtain (*h*_*f*_), equation (18), which is dimensionless, a Nusselt number was used, where (*K*_*f*_) represents the thermal conductivity of the heat transport fluid of the water. The values of the thermal conductivity were obtained according to the correlations described in [19].

(18) $$\begin{array}{c} \text{Nu}=\frac{h_{f}D}{K_{f}}. \end{array}$$

Likewise, (Nu) can be obtained through the Petukhov equation (19) [20]:

(19) $$\begin{array}{c} \text{Nu}=\frac{\left(F/8\right)\text{Re Pr}}{1,07+12,7{\left(F/8\right)}^{0,5}\left({\text{Pr}}^{\left(2/3\right)}-1\right)}. \end{array}$$

The friction factor (F), given by equation (20), is a valid relationship for the fully developed turbulent regime (3000 < Re < 5 × 10^6^), where the Reynolds number is calculated by equation (21), in which (*ρ*), (*v*_*f*_), and (*D*_*i*_) represent the fluid density, flow velocity, and internal diameter of the absorber tube, respectively.

(20) $$\begin{array}{c} F={\left(0,75{\text{lnRe}}^{1,64}\right)}^{-2}, \end{array}$$

(21) $$\begin{array}{c} \text{Re}=\frac{dv_{f}D_{i}}{\mu }, \end{array}$$

(22) $$\begin{array}{c} \text{Pr}=\frac{\mu Cp}{K_{f}}. \end{array}$$

#### 1.2.1. Determining Errors

The error propagation analysis is necessary to determine the error in the efficiency measure. Considering equation (4) where the expanded uncertainty corresponds to (*ε*_*η*_), the yield error is calculated using equation (23):

(23) $$\begin{array}{c} \frac{\varepsilon_{\eta }}{\eta }=\sqrt{{\left(\frac{\varepsilon_{\dot{m}}}{\dot{m}}\right)}^{2}+{\left(\frac{\varepsilon C_{p}}{C_{p}}\right)}^{2}+{\left(\frac{\varepsilon \Delta T}{\Delta T}\right)}^{2}+{\left(\frac{\varepsilon I_{b}}{I_{b}}\right)}^{2}+{\left(\frac{\varepsilon A_{a}}{A_{a}}\right)}^{2}.\;}\; \end{array}$$

Thus, the expanded uncertainty of each element of equation (4) is presented in Table 2. The probability distribution obtained with equation (23) is Gaussian even if the inputs do not have the same distribution.

The uncertainty attributed to *C*_*p*_ was related according to equation (24), where *T*_*m*_=(*T*_*o*_ − *T*_*i*_)/2, considering the physical properties of water described by [19].

(24) $$\begin{array}{c} \varepsilon C_{p}=\frac{\partial C_{p}}{\partial T_{m}}\varepsilon \Delta T. \end{array}$$

## 2. Results and Discussion

The performance of the collector was evaluated using the equation (4). For this, the temperature variation data are measured, shown in Figure 7(a), and the mass flow of the fluid is measured using the flow sensor shown in Figure 7(b), and direct solar radiation was obtained from atlas Solametric of the State of Paraná [21].

**Figure 8:**
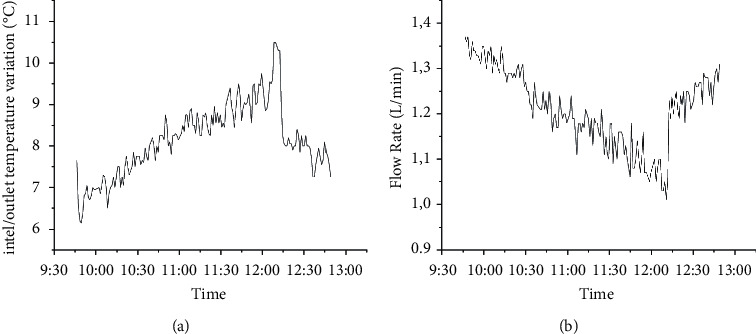
Graph with PTSC operating data: (a) temperature and (b) flow rate variation for measurements carried out on 10/13/2021.

As shown in Figure 8, the time required for heating the proposed collector was 4.5 minutes, thus reaching 63.2% of the temperature variation between the beginning of the test and the stabilization of the outlet temperature.

**Figure 9:**
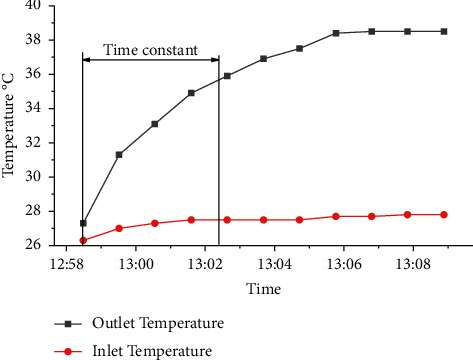
Evaluation of collector heating time.

Using the data obtained experimentally and presented in Figure 8, it was possible to determine the theoretical and experimental efficiency of the collector for which equations (4) and (5) were applied, resulting in the graph in Figure 9.

**Figure 10:**
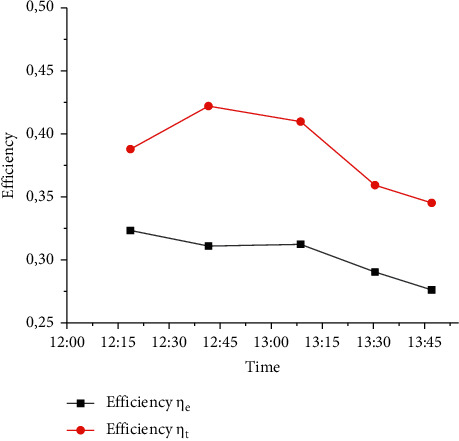
Comparison between theoretical and experimental efficiency.

**Figure 11:**
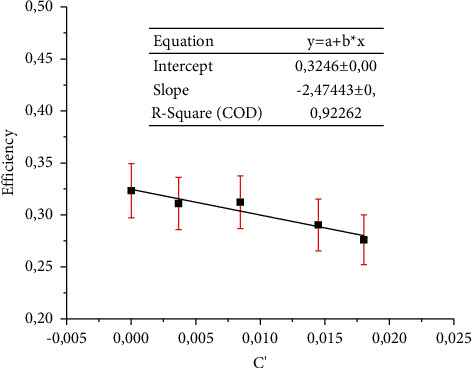
The collector efficiency as a function of temperature variation *C*′ and the error bars plotted in this graph represent the standard deviation according to Table 3.

Another way to determine the thermal efficiency is using equation (10). Thus, the graph in Figure 10 presents the thermal efficiency for different fluid inlet temperatures in relation to the ambient temperature by the direct solar radiation available (*c*′), so the efficiency is described according to the linear relationship: *η*=0,3246 − 2,4744*c*′ (see Figure11).

Figure 11 shows that there is an efficiency loss due to the increase of the parameter (*c*′). That is, with the increase of the inlet temperature, there is a reduction in the efficiency of this collector because the solar radiation was considered fixed at 950 W/m^2^ following the methodology proposed in [22]. Finally, the average error (*ε*_*η*_) was 0.039 and the maximum error (*ε*_max_) was 0.042 using which it was possible to plot the error of each measurement in the graph in Figure 11.

**Figure 12:**
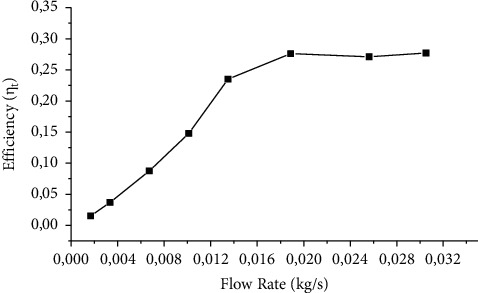
Efficiency as a function of mass flow.

The collector proposed also had its efficiency evaluated as a function of the mass flow of the fluid, resulting in the graph in Figure 12, where it is possible to observe that the efficiency is maximum when the flow operates under turbulent conditions.

Finally, it is noteworthy that the optical efficiency of the collector was determined using equation (7), where the removal factor (*F*_*r*_) was obtained according to equation (11). Thus, the optical efficiency of the collector is *η*_*o*_ = 0.41. The collector performance parameters are shown in Table 3.

The performance of the proposed collector can also be compared with other similar devices as shown in Table 4, in which it is possible to verify that the efficiency of the proposed collector is below the analyzed collectors. It is mainly due to the low optical efficiency and the heat losses due to the lack of a glass envelope over the collection tube.

## 3. Conclusions

The time required for the PTSC to reach thermal equilibrium is 4.5 minutes, which allows the equipment to be evaluated shortly after being put into operation.

Thermal efficiency was obtained below the efficiency found in similar collectors for the proposed PTSC. This difference can be attributed mainly to two characteristics of the collector: the first is the lack of the glass envelope on the collector tube, which provides a greater thermal loss by convection, and this loss is directly related to environmental conditions, such as speed wind and ambient temperature; and the second condition that can impair thermal efficiency is the type of coating used on the absorber tube, which has a high emissivity, allowing a portion of the absorbed heat to be lost in the form of thermal radiation. Therefore, for future development, it is recommended to use an evacuated glass envelope around the absorber tube (concentrically) and the use of a selective cover to improve the performance of the PTSC.

In the case of optical efficiency, it presents values lower than expected when compared with similar systems. This may be because the reflective surface is partially diffused due to the roughness of the steel plate used as a base, increasing the error of approximately 25% image formation in the system. The other variables such as the constructive geometric precision and the solar tracking system presented a good performance.

The solar tracking system performs well, keeping the tracking error lower than the angle of acceptance of the parabola reflection in the absorber tube.

The model applied in the analytical part considers the parameters calculated with the efficiency equation obtained experimentally, so the error among the values found is mainly because it does not have real data on direct solar radiation and the wide distribution of values of efficiency as a function of the inlet temperature makes the linear relationship obtained less precise, contributing to the increase of this error.

When compared with similar systems, the proposed collector has lower efficiency, which is related to the improvement of the items mentioned above (optical efficiency and heat loss).

Regarding the didactic application, it was possible to use the collector with minimal supervision and with great reliability because of the low cost of construction combined with the accurate tracking systems and the easy visualization of the data through the supervisory system.

Thus, for future development, the use of an evacuated glass envelope over the absorber tube and a reflective surface with better reflectivity indices should improve the overall performance of the collector. Also, a pyrheliometer of normal incidence was installed to obtain more precise data on the incident direct radiation for a better precision analysis. And finally, the production of experimental teaching procedures should make teaching practice easier for the teacher.

In future studies, a computational analysis using CFD (computational fluid dynamics) can be performed to visualize and quantify heat transfer losses for design improvements.

## Figures and Tables

**Table 1:** PTSC geometric parameters.

Parameter	Dimension
Parabolic trough opening (*w*_*a*_)	110 cm
Parabolic trough length (L)	200 cm
Collector opening area	2, 2 m^2^
Geometric concentration factor (C)	8, 34
Opening angle (*φ*_*r*_)	120°
Collector opening at focal height (*Hp*)	32, 88 cm
Length of the parabola curve (*S*)	140 cm
Focal length (*f*_*L*_)	16 cm

**Table 2:** Values of expanded uncertainties.

Greatness	Representation	Standard deviation	Distribution
Mass flow	$\varepsilon_{\dot{m}}$	0,001	Gaussian
*C*_*p*_	*εC*_*p*_	See equation (24)	Gaussian
Temperature variation	*ε*Δ*T*	0,5	Gaussian
Collector area	*εA*_*a*_	0,01	Uniform
Direct radiation	*εI*_*b*_	Constant	Uniform

**Table 3:** Experimental values for collector operation.

Parameter	Value	Notes
Removal factor (*F*_*r*_)	0,84	Equation (11)
Optical efficiency (*η*_0_)	0,41	Equation (6)
Thermal loss coefficient (*U*_*L*_)	21,7	Equation (14)
Ambient temperature	25°C	Average
Direct radiation (*I*_*b*_)	950 W/m^2^	Estimated

**Table 4:** Comparison of thermal efficiency among different collectors.

Author	Efficiency (%)	Optical efficiency (%)	**U**_**L**_(**Wm**^−**2**^°**C**^−**1**^)	**F**_**r**_
Torres et al. [23]	52,38 (max)	NA	NA	NA
Coccia et al. [18]	65,00 (max)	66,80	6,42	0,985
Jaramillo et al. [24], PTC45	38,00 (max)	48,00	43,09	0,90
Jaramillo et al. [24], PTC90	67,00% (max)	70,00	34,98	0,90
Proposed collector	34,38% (max)	41,00	21,7	0,84

## Data Availability

The experimental data used to support the findings of this study are available from the corresponding author upon request.
